# Dissecting a neuron network: FIB-SEM-based 3D-reconstruction of the visual neuropils in the sea spider *Achelia langi* (Dohrn, 1881) (Pycnogonida)

**DOI:** 10.1186/s12915-014-0059-3

**Published:** 2014-08-13

**Authors:** Tobias Lehmann, Martin Heß, Gerhard Wanner, Roland R Melzer

**Affiliations:** Bavarian State Collection of Zoology – SNSB, Münchhausenstraße 21, 81247 Munich, Germany; Department Biology II, Ludwig-Maximilians-Universität München, Großhaderner Straße 2, 82152 Planegg-Martinsried, Germany; Department Biology I, Ludwig-Maximilians-Universität München, Großhaderner Straße 2-4, 82152 Planegg-Martinsried, Germany; GeoBio-Center LMU, Richard-Wagner-Straße 10, 80333 Munich, Germany

**Keywords:** Median eyes, Lateral eyes, Visual system, Connectome, Arthropoda, Chelicerata, Pycnogonida

## Abstract

**Background:**

The research field of connectomics arose just recently with the development of new three-dimensional-electron microscopy (EM) techniques and increasing computing power. So far, only a few model species (for example, mouse, the nematode *Caenorhabditis elegans*, and the fruit fly *Drosophila melanogaster*) have been studied using this approach. Here, we present a first attempt to expand this circle to include pycnogonids, which hold a key position for the understanding of arthropod evolution. The visual neuropils in *Achelia langi* are studied using a focused ion beam-scanning electron microscope (FIB-SEM) crossbeam-workstation, and a three-dimensional serial reconstruction of the connectome is presented.

**Results:**

The two eyes of each hemisphere of the sea spider’s eye tubercle are connected to a first and a second visual neuropil. The first visual neuropil is subdivided in two hemineuropils, each responsible for one eye and stratified into three layers. Six different neuron types postsynaptic to the retinula (R-cells) axons are characterized by their morphology: five types of descending unipolar neurons and one type of ascending neurons. These cell types are also identified by Golgi impregnations. Mapping of all identifiable chemical synapses indicates that the descending unipolar neurons are postsynaptic to the R-cells and, hence, are second-order neurons. The ascending neurons are predominantly presynaptic and sometimes postsynaptic to the R-cells and may play a feedback role.

**Conclusions:**

Comparing these results with the compound eye visual system of crustaceans and insects – the only arthropod visual system studied so far in such detail – we found striking similarities in the morphology and synaptic organization of the different neuron types. Hence, the visual system of pycnogonids shows features of both chelicerate median and mandibulate lateral eyes.

**Electronic supplementary material:**

The online version of this article (doi:10.1186/s12915-014-0059-3) contains supplementary material, which is available to authorized users.

## Background

One of the most intriguing questions in vision research is how the neuronal circuitry processes the visual input from the photoreceptors, that is, the neuronal correlate of the eye and retina’s visual architecture. Cell-type-specific wiring rules, the divergence and convergence of information channels and the maintenance of retinotopy are some of the core issues. Here, data acquisition entails the challenge of covering volumes of thousands of cubic micrometers (to enclose entire neurons) with a voxel-resolution of only a few nanometers (to correctly trace membrane profiles and to see synaptic structures). One promising approach is (three-dimensional) reconstruction from serial section transmission electron microscopy (TEM), which is nowadays a well-established way of analyzing circuitry of neural networks [1–3]. However, several hundreds of sections or even more have to be cut without any loss of sections, inspected and photographed with the TEM, resulting in an enormous data volume, which is followed by a complex elastic alignment to compensate inevitable image distortions using an elastic alignment program (for example, TrakEM2 [4,5]). Hence, the main criterion in selecting a suitable subject for such a study is a small size. In analyzing nervous systems regarding connectomics, either small animals with a small central nervous system (CNS) or a restricted region within the CNS or even within a particular neuropil are possible study subjects to obtain a comprehensive data stack.

Early serial section EM research dealing with arthropod visual systems was performed by Macagno *et al*. [6] in analyzing the visual system in *Daphnia magna* and later by Meinertzhagen and O'Neil [7] in reconstructing synaptic connections in the lamina cartridges of *Drosophila*. A classic example for the reconstruction of a whole nervous system is the nematode *Caenorhabditis elegans* [8,9]. An early attempt to use computerized three-dimensional reconstructions to study the axonal wiring of photoreceptor axons is that by Melzer *et al*. [10] in midges and the scorpion fly. These studies did not have today’s computing power at their disposal. In the last few years, personal computers have become capable of handling the enormous data volumes inevitable for three-dimensional reconstructions from serial section TEM. Previous studies using this power have focused on the lamina and medulla in the fruit fly *Drosophila melanogaster* [11–13].

Furthermore, in recent years, a new generation of three-dimensional-EM tools has been developed [14–16], which includes Serial Block Face Scanning Electron Microscopy (SBF-SEM or simply SBEM) based either on mechanical sectioning [17,18] or milling with a focused ion beam (FIB-SEM, [19,20]). These methods enhanced the potential of three-dimensional-EM considerably and are applied, for example, on nervous tissue [21–23] and to display and count synapses in vertebrates [24–26].

In the present study, we analyze the visual neuropils in the pycnogonid *Achelia langi* with one of these methods, namely FIB-SEM. The advantages of this cutting-edge method are that compared to serial section TEM, the generation of the image-stack is much faster and without loss, the images are perfectly aligned with a z-resolution down to 5 nm (TEM approximately 70 nm), and the x-y-resolution and contrast compared to TEM are only slightly reduced.

The Pycnogonida, or sea spiders, are exclusively marine invertebrates, numbering more than 1,300 species worldwide [27]. Although largely unnoticed due to their cryptic life habits and economic insignificance, sea spiders are common benthic animals occurring from the littoral zone to the deep sea, from tropical to polar waters. The fossil record dates back to the early Paleozoic Era, with the earliest unequivocal records dating back to the Ordovician and Silur [28,29]. It has even been hypothesized that Pycnogonida might date back to the Cambrian [30]. The phylogenetic position of the Pycnogonida has long been controversial and is still under debate. Pycnogonids are placed either within the Chelicerata as sister taxon of the Euchelicerata or as sister taxon of all other Euarthropoda [31,32]. In recent years the debate has shifted in favor of sea spiders being chelicerates [33,34]. Studies of the development and the innervation patterns of the brain have shown that characters from the nervous system can contribute important arguments to the discussion about the phylogenetic position of sea spiders [35–39].

For this field of research in general, comparing the structure and development of nervous systems in a phylogenetic context, two different approaches have been established: ‘neurophylogeny’ [40,41] and ‘neural cladistics’ [42,43].

The sensory parts of the arthropod protocerebrum are primarily responsible for the visual system. Two different types of eyes are found in arthropods, median and lateral eyes. Pycnogonids possess only a periscope-like ocular tubercle with four little eyes or ocelli (*sensu* Richter *et al*. [44]) generally interpreted as median eyes, whereas classical lateral eyes are absent. Studies using light [45,46] and electron microscopy [47,48] have revealed that these pigment cup eyes have a cuticular lens and a latticed rhabdom surrounded by pigment layers, features typical of median eyes. Derived conditions might include the structure of the retinula or R-cells, described as ‘pseudoinverted’ [48], and the presence of a tapetum lucidum (guanine multilayer reflector). The connection of these R-cells to the brain was recently analyzed with classical and modern neuroanatomical techniques to identify the visual neuropils [49]. Hence, the pycnogonid visual system is composed of a thickening dorsolateral to the protocerebrum where the nerve fibers from the two eyes of one hemisphere concentrate, a bifurcated visual tract and two successive distinct visual neuropils. This innervation pattern is very similar to that of the eyes in *Euperipatoides rowelli* (Onychophora) [50] and the median rudimentary eye in *Limulus polyphemus* (Xiphosura) [51,52].

The architecture of the visual system of sea spiders is relatively simple compared to that of many other arthropods. Considering the phylogenetic position of pycnogonids as basal chelicerates or even arthropods suggested by both tree reconstruction and the fossil record, one can conclude that the selection of Pycnogonida allows us to understand a visual system in a detailed way due to its simplicity and to learn more about eye evolution in arthropods.

In the present study, we take a closer look at the visual neuropils in the pyconogonid *Achelia langi* (Ammotheidae) using the advantages of FIB-SEM. In a low-resolution stack, the arrangement of the visual nerve fibers and neuropils is analyzed. In a second, medium-resolution stack, neurons postsynaptic to the R-cells are three-dimensionally reconstructed to gain a more detailed view of the neuroanatomy of the pycnogonid visual system. To utilize two strains of evidence, the morphology of these cells is additionally compared to Golgi-impregnated profiles in *Achelia vulgaris*. Finally, in a third high-resolution stack, the distribution of synapses within these cells is analyzed. These findings reveal features of the visual system generally studied in Arthropoda to allow comparisons with other lineages.

## Results

### General layout of the visual neuropils in the protocerebrum

**Figure 1:**
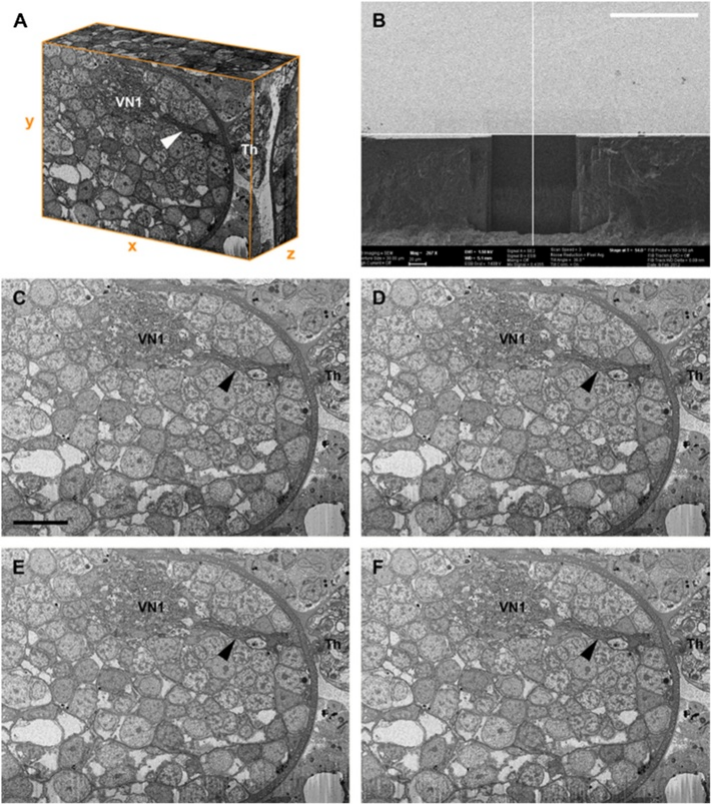
**Pycnogonid visual neuropils studied with focused ion beam SEM technique. A,** three-dimensional volume of low-resolution image stack; note sharp xz- and yz-projections due to almost perfect alignment of FIB-SEM. **B,** backscattered electron image of mesa at beginning of milling by FIB-SEM. Bar 100 μm. **C–F,** short consecutive image series at beginning of stack; note minor but visible structural change from slice number 80 (C) to slice number 83 (F). Bar 10 μm. Arrowhead, visual tract projecting through cell body rind; Th, thickening; VN1, visual neuropil 1. SEM, scanning electron microscopy.

**Figure 2:**
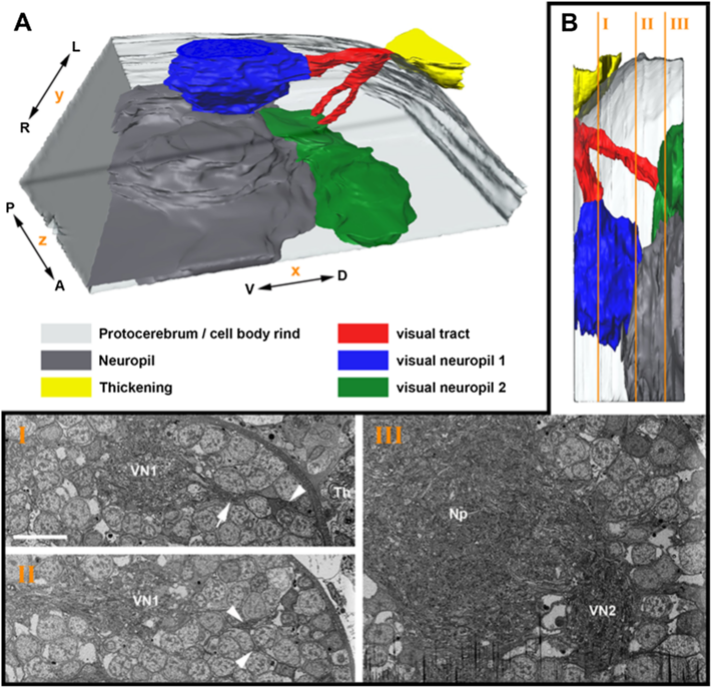
**Three-dimensional serial reconstruction of visual neuropils of left hemisphere in** ***Achelia*** ***langi*** **on basis of low-resolution image stack. A,** three-dimensional reconstruction showing the arrangement and orientation of neuropils; posterior is up, dorsal is right. **B,** three selected sections showing original data for reconstruction; position of sections indicated in three-dimensional reconstruction top right. **I** ***,*** medium range of visual neuropil 1 (slice number 125); note two subsets of visual tract projecting through cell body rind, arrow indicating subset projecting to visual neuropil 1, arrowhead indicating subset projecting to visual neuropil 2. **II,** low range of visual neuropil 1 (slice number 376); note two subsets of visual tract projecting through cell body rind to visual neuropil 2 (arrowheads). **III,** beginning of visual neuropil 2 (slice number 640). Bar 10 μm. A, anterior; D, Dorsal; L, left; Np, neuropil; P, posterior; R, right; Th, thickening; V, ventral; VN1, visual neuropil 1; VN2, visual neuropil 2.

In the examined area of the low-resolution FIB stack, the visual tract bifurcates. After entering the protocerebrum, one part of the fibers projects to the first visual neuropil located dorsolaterally in the anterior part of the protocerebrum as an ovoid region laterally embedded in the cell body rind of the brain (Figures 1, 2). The other part of the fibers projects to the second visual neuropil. These fibers likewise bifurcate and enter the second visual neuropil in two portions. This neuropil is located deeper, under the cell body rind and in a more anterior and central position in the protocerebrum (Figure 2). Both neuropils are in contact with the rest of the neuropils of the protocerebrum. The posterior part of the first visual neuropil is ventrally connected to the neuropil of the protocerebrum. The second visual neuropil, in turn, is posteriorly not clearly separated from the remaining neuropils (Figure 2).

### Cell types in the first visual neuropil

**Figure 3:**
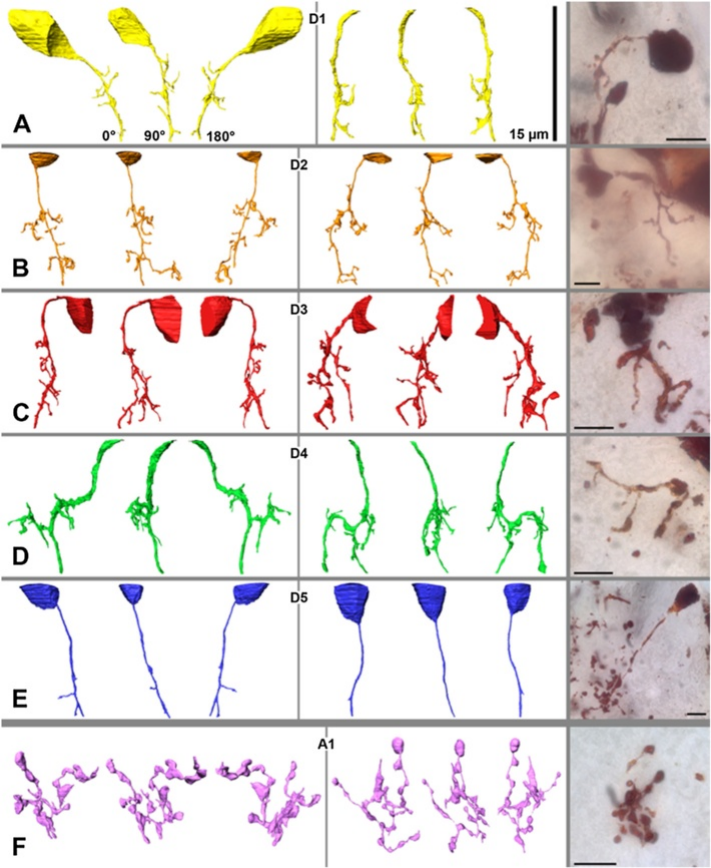
**Profiles of six different cell types found in 3D-reconstructions and Golgi-preparations** **.** All profiles were reconstructed from visual neuropil 1 of right hemisphere in Achelia langi on the basis of medium-resolution image stack. Two representatives of each cell type are shown at three different angles; note additional corresponding profiles of Golgi-impregnated cells on right-hand side. **A,** Descending unipolar neuron 1 (D1), characterized by unbranched neurite with several collaterals. **B,** Descending unipolar neuron 2 (D2), characterized by branched neurite with several collaterals. **C,** Descending unipolar neuron 3 (D3), characterized by bifurcation of neurite with several collaterals. **D,** Descending unipolar neuron 4 (D4), characterized by h-shaped neurite with each branch reaching into one hemisphere; with several collaterals as well. **E,** Descending unipolar neuron 5 (D5), characterized by unbranched neurite without or with just a few collaterals. **F,** Ascending unipolar neuron 1 (A1), characterized by neurite with multiple branches, each with several large boutons and thin connectors in between. Each cell spreads throughout wide reaches of both hemineuropils.

**Figure 4:**
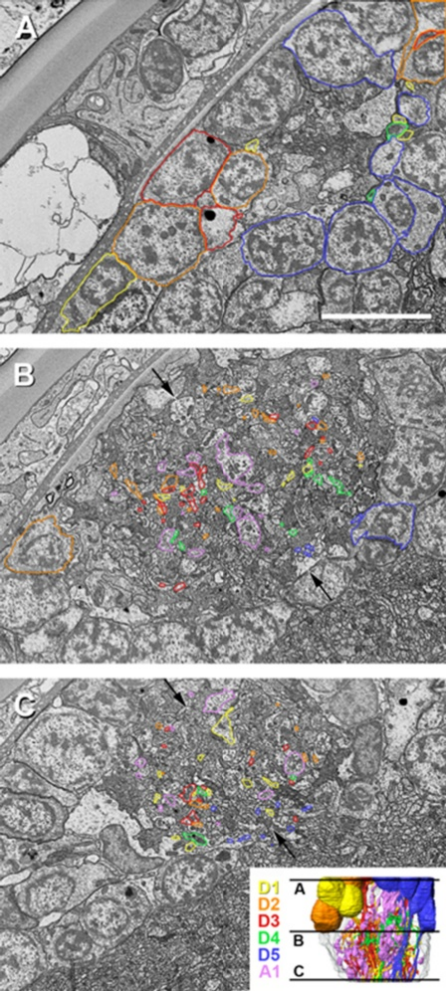
**Three selected sections with labeling of different cell types showing original data for reconstruction.** Position of sections indicated in three-dimensional reconstruction bottom right; note cells with high electron density identified as retinula axon terminals surrounded by cells with low electron density identified as postsynaptic neurons. **A,** beginning of visual neuropil 1 (slice number 23); neuropil surrounded by cell bodies of descending unipolar neurons. Bar 5 μm. **B,** medium range of visual neuropil 1 (slice number 523); arrows indicate subdivision of neuropil into two hemineuropils. **C,** low range of visual neuropil 1 (slice number 1,017); arrows indicate subdivision of neuropil.

**Figure 5:**
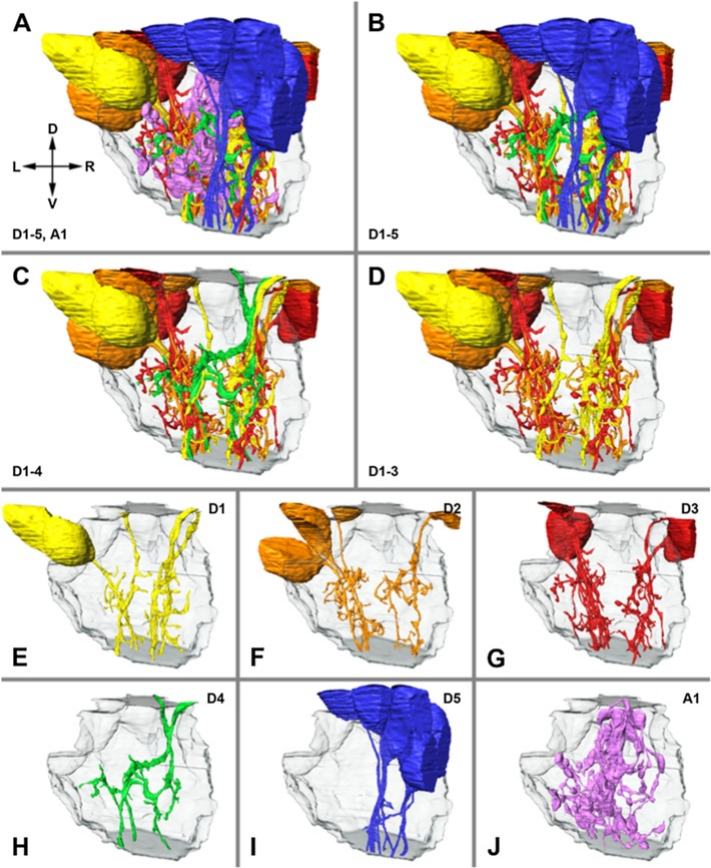
**Lateral view of three-dimensional reconstruction of visual neuropil 1. A,** all reconstructed cells of all six neuron types shown; dorsal is up. **B,** A1 neurons omitted, thus subdivision of neuropil becomes visible; note D5 neurons mainly in right hemineuropil. **C,** A1 and D5 neurons omitted; note D4 neurons occur in both hemineuropils at once. **D,** A1, D4 and D5 neurons omitted; note D1, D2 and D3 neurons build two hemineuropils. **E–J,** distribution of different cell types separately within neuropil. D, dorsal; L, left; R, right; V, ventral.

**Figure 6:**
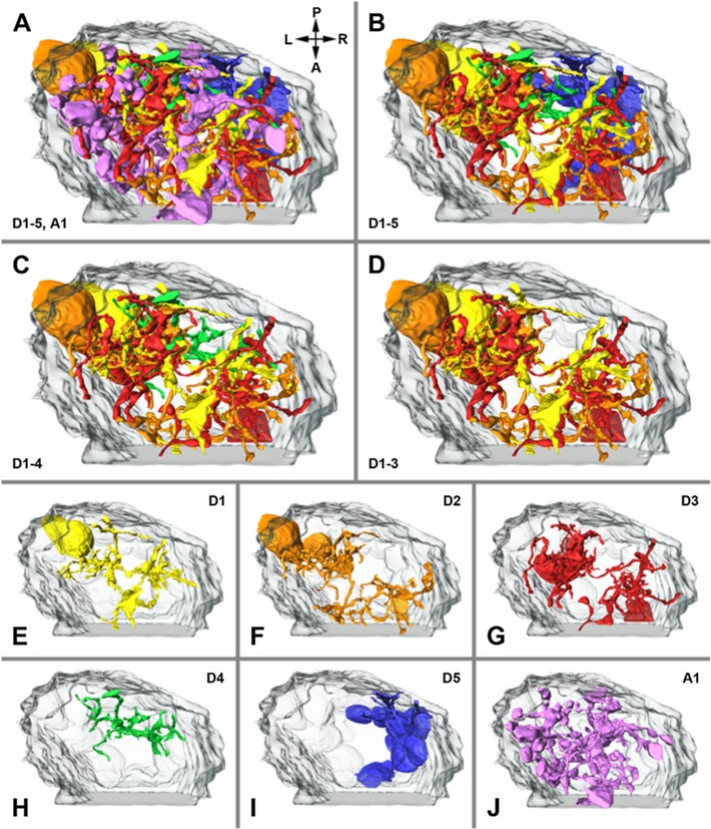
**Three-dimensional reconstruction of visual neuropil 1 viewed from bottom up. A**, all reconstructed cells of all six neuron types shown; posterior is up. **B,** A1 neurons omitted, thus subdivision of neuropil becomes visible; note D5 neurons mainly in right hemineuropil. **C,** A1 and D5 neurons omitted; note D4 neurons occur in both hemineuropils at once. **D,** A1, D4 and D5 neurons omitted; note D1, D2 and D3 neurons build two hemineuropils. **E–J,** distribution of different cell types shown separately within neuropil. A, anterior; L, left; P, posterior; R, right.

In the FIB-SEM (medium-resolution stack) based examination of *A. langi,* a division of the first visual neuropil into two equal subunits or hemineuropils (see also below) was observed (Figures 3, 4, 5 and 6). This division appears in the distal third of the neuropil and is apparent throughout the rest of the neuropil. In the FIB-SEM images, the two hemineuropils are characterized by neurites, mostly of small diameters, and are divided primarily by bulky neurites with larger diameters (Figures 4B, C).

**Figure 7:**
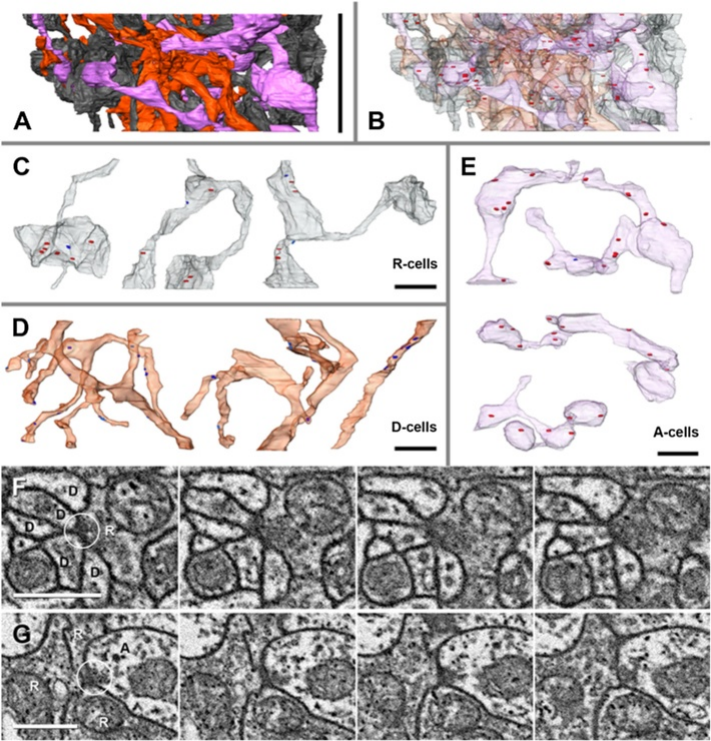
**Three-dimensional serial reconstruction of medium range of visual neuropil 1 of right hemisphere in** ***Achelia langi*** **based on high resolution image stack. A,** all reconstructed cells of all three neuron types (R-, D- and A-cells) shown. Bar 3.2 μm (that is, z-range of the stack). **B,** all reconstructed cells of all three neuron types (R-, D- and A-cells) shown in transparent and all chemical synapses (presynaptic vesicle clusters) found within these cells indicated in red. **C,** Profiles of three different R-cells; presynaptic sites indicated in red, postsynaptic sites indicated in blue. Bar 1 μm. **D,** Profiles of three different D-cells; postsynaptic sites indicated in blue, no presynaptic sites in these cells. Bar 1 μm. **E,** Profiles of three different A-cells; presynaptic sites indicated in red, postsynaptic sites indicated in blue. Bar 1 μm. **F,** Series of four consecutive FIB-SEM images showing a synapse (encircled) between R- and D-cells (slice numbers 26 to 29): about five D-cells (cells with low electron density) postsynaptic to one R-cell (cell with high electron density). Bar 500 nm. **G,** Series of four consecutive FIB-SEM images showing a synapse (encircled) between A- and R-cells (slice numbers 27 to 30): three R-cells (cells with high electron density) postsynaptic to one A-cell (cell with low electron density). Bar 500 nm. FIB-SEM, focused ion beam-scanning electron microscopy.

Furthermore, six different types of neurons were reconstructed and classified on the basis of their morphology: five descending cell types (Figures 3A to E) and one ascending cell type (Figure 3F). All of these neurons can also be identified by Golgi impregnations (Figure 3 rightmost). The descending cells are unipolar neurons with cell bodies in the cell body rind above the neuropil, which send a single neurite each into the first visual neuropil. (To keep the Results section free from homology assumptions, the term ‘monopolar cells’ is intentionally avoided because this term is occupied by the monopolar cells in the compound eye visual system in Pancrustacea; see Discussion). Most of the descending neurons can be traced from the cell body all the way through the neuropil to the end of the image stack. Neurons reconstructed without cell bodies can be allocated to their particular cell type on the basis of the morphology of the neurites. A classification of the ascending neurons cannot be made because the cell bodies of these cells are beyond the examined area. However, the cell bodies must be located below the neuropil, whereas the neurites end before the top end of the neuropil. A large section of the ascending cells and all of the descending cells with cell bodies within the examined volume above the neuropil are reconstructed, and some cells are allocated due to their neurite morphology. Individual retinula axons (cells with a high electron density), due to the low contrast of these cells in the FIB-SEM images, and synapses, due to the too-low resolution, cannot be reliably traced in the medium-resolution stack. In the stack having the highest resolution, however, the R-cells and synapses are reconstructed (see below and Figure 7). The total volume of interest (that is, neuropil and cell bodies in the examined area) is approximately 4,800 μm^3^, and the volume of all reconstructed cells is 567 μm^3^; hence, the reconstructed cells occupy approximately 12% of the volume.

### Descending unipolar neurons (D1 to D5)

#### D1 (Figures 3A, 4; n = 6)

The cell bodies in two of the six cells could be reconstructed; the remaining cells were allocated due to their neurite morphology. Cell bodies are found in the cell body rind above or lateral to the upper third of the neuropil. The neurites are unbranched and slightly curved. All cells can be traced to the end of the image stack. Short collaterals occur in tangential and radial directions throughout the neurite but are accumulated in the medium range of the neuropil. Each cell profile covers only a small area of the neuropil. D1 neurons can be found throughout the neuropil, whereas a single neuron is restricted to only one hemineuropil.

#### D2 (Figures 3B, 4; n = 5)

The cell bodies could be at least partially reconstructed in all cells. They are found in the cell body rind above or lateral to the upper third of the neuropil. The neurites are branched and slightly curved. The branching always occurs in the medium range of the neuropil, and the neurite is divided into a short and a long branch. The long branch of all cells can be traced to the end of the image stack; the short branch ends in the medium range of the neuropil and is radially oriented. Short collaterals occur in tangential and radial directions throughout both branches of the neurite. Each cell profile covers a larger area of the neuropil compared to the D1 cells. D2 neurons can be found throughout the neuropil, whereas a single neuron is restricted to only one hemineuropil.

#### D3 (Figures 3C, 4; n = 6)

The cell bodies could be at least partially reconstructed in four cells; the remaining cells were allocated due to neurite morphology. The cell bodies are found in the cell body rind above or lateral to the upper third of the neuropil. The neurites are bifurcated. The bifurcation always occurs in the medium range of the neuropil. Both branches can be traced to the end of the image stack. Short collaterals occur in tangential and radial directions throughout both branches of the neurite. Similarly to the D2 cells, each cell profile covers a larger area of the neuropil compared to the D1 cells. D3 neurons can be found throughout the neuropil, whereas a single neuron is restricted to only one hemineuropil.

#### D4 (Figures 3D, 4; n = 2)

One cell could be reconstructed with only a small portion of the cell body; the other cell was allocated due to the neurite morphology. The cell bodies are found in the cell body rind above the neuropil. The neurites are h-shaped. In the medium range of the neuropil, the neurite is radially oriented and builds two tangential branches, each reaching into one hemineuropil. Short collaterals occur in tangential and radial directions throughout the neurite. All cells can be traced to the end of the image stack. A single D4 neuron occurs in both hemineuropils at once. The cell profiles cover, compared to the other D cells, the largest area of the neuropil because they occur in both hemineuropils.

#### D5 (Figures 3E, 4; n = 9)

The cell bodies could be reconstructed at least partially in all cells. They are found in the cell body rind above or lateral to the upper third of the neuropil. The neurites are unbranched, straight or only slightly curved. Six neurons are without any collaterals and three neurons with just one or two short tangential collaterals. All cells can be traced to the end of the image stack. D5 neurons can be found in the right hemineuropil only. These neurons cross the right hemineuropil at its edge, and in the lower part of the neuropil they can be found in the area that divides the two hemineuropils.

### Ascending neurons (A1)

#### A1 (Figures 3F, 4; n = 6)

Cell bodies were not found in the examined area. All reconstructed cells end in the upper third of the neuropil; hence, the neurites could not be traced from the most proximal slice throughout the neuropil to the distal end. The cell bodies of these neurons must, therefore, be located below the neuropil, meaning that these cells are ascending neurons. The neurites are equipped with multiple branches, each with several large boutons or varicosities and thin connectors in between. These cells have a high-turgor appearance; this means that the boutons have rounded contours. A1 neurons can be found throughout the neuropil; however, branches of A1 neurons accumulate in the area that divides the two hemineuropils. A single neuron occurs in both hemineuropils at once. Each cell profile covers a large area of the neuropil.

### Organization of the first visual neuropil

When all neuron types (D1 to D5, A1) are shown together, no special organization of the neuropil is identifiable (Figures 5A; 6A; Additional file 1). However, by removing the A1 neurons from the three-dimensional reconstruction, a subdivision of the visual neuropil becomes apparent (Figures 5B; 6B), which is also observed in the FIB-SEM images (see above and Figures 4B, C). The neuropil is divided into two hemineuropils of equal size. Between the hemineuropils, a border zone exists where fewer of the D-cells occur. While the D1 to D4 cells are evenly distributed in both hemineuropils, the D5 cells occur only in the right hemineuropil (Figures 5B; 6B). When the D5 cells are removed from the reconstruction (Figures 5C, D; 6C, D) the subdivision becomes more obvious; moreover, a feature of the D4 cell becomes visible: these neurons connect the two hemineuropils. Whereas just a few collaterals of the D1 to D3 cells reach into the border zone, branches of the D4 cells run through this border and connect both hemineuropils.

When each cell type is shown on its own, their characteristic features become visible (Figures 5E–J; 6E–J). The D1, D2 and D3 cells form the main body (apart from the A1 cells) of the visual neuropil (Figures 5D–G; 6D–G); these cells form the two hemineuropils. Just a few collaterals, but not the main branches, of the D1, D2 and D3 cells of the two hemineuropils reach into the border zone in between. In contrast to the D1, D2 and D3 cells, the main branches of the D4 cells cross the border zone and occur in both hemineuropils at once (Figures 5H; 6H). The D5 cells take a special position; these cells were found in the examined area only in the right hemineuropil (Figures 5I; 6I). The neurites of the D5 cells run along the posterior edge of this hemineuropil, and in the lower part they are found primarily in the border zone between the hemineuropils.

The A1 cells can be distinguished from the D1 to D5 cells in morphology and distribution. These cells do not form two hemineuropils; rather, the neurites of these cells are distributed throughout the neuropil and are accumulated in the border zone of the two hemineuropils.

### Synaptic organization of the first visual neuropil

In the stack with the highest resolution, R-cells as well as synapses can be reconstructed in addition to descending and ascending neurons (Figure 7). The stack is located in the medium range of the neuropil. Cells of one hemineuropil were reconstructed in which three different cell types are allocated on the basis of their neurite morphology: R-cells, D-cells and A-cells. Ultrastructurally, chemical synapses can be recognized by a presynaptic concentration of electron-dense vesicles and electron-dense material in the synaptic cleft accompanied by high membrane density (Figures 7F, G). However, postsynaptically, no special synaptic structures are found. In the investigated volume, no sign of electric synapses (for example, gap junctions) could be detected. Altogether, 95 chemical synapses are identified in the studied volume. These are often multiple-contact synapses (dyads, triads, tetrads, and so on). Altogether, approximately 13% of the cells in the hemineuropil are reconstructed (approximately 260 cells counted in the field of interest in the first slice, 33 cells reconstructed). The total volume of interest (area of the examined hemineuropil) is approximately 260 μm^3^ and the volume of all cells reconstructed is 34 μm^3^; hence, these cells occupy 13% of the volume.

#### R-cells (Figure 7C; n = 18)

This cell type could not be reconstructed in the medium-resolution stack but could in the high-resolution stack. In the FIB-SEM images, these cells are characterized by high electron density. The morphology of R-cells is similar to that of A-cells: the neurites have multiple branches, each with several large boutons or varicosities and thin connectors in between, with the difference that the R-cells have a low-turgor appearance. This means that the shape of these cells adapts to the shape of the surrounding cells and the boutons have limp contours. Within these cells, an average of 3.3 synapses per cell was found in the reconstructed area; these occur primarily in the boutons. R-cells are predominantly presynaptic to D-cells and sometimes to A-cells. Furthermore, R-cells are frequently postsynaptic to A-cells (Table 1). One individual R-cell is presynaptic to several D-cells.

**Table 1:** **Synaptic pattern of the different cell types in the high-resolution stack**

**Presynaptic cells**			**→ presynaptic to ↓**
**R-cells (number = 18)**	**D-Cells (number = 7)**	**A-cells (number = 8)**	
0	1	43	R-cells
32	1	8	D-cells
5	1	0	A-cells
22	3	14	cells not reconstructed
3.3	0.9	8.1	synapses/cell (average)^a^

^a^in the reconstructed volume.

#### D-cells (Figure 7D; n = 7)

These cells were allocated, due to their neurite morphology, to the D-cells of the medium-resolution stack (Figure 3). A subdivision into the five different D-cell types cannot be made because only a small portion of the cells on the z-axis were reconstructed. Within these cells, just a few areas with increased vesicle density and other indicators of presynaptic activity were found in the reconstructed area; most cells are without such presynaptic sites. D-cells are predominantly postsynaptic to R-cells and sometimes to A-cells (Table 1). One individual D-cell is postsynaptic to several R-cells.

#### A-cells (Figure 7E; n = 8)

These cells were allocated, due to their neurite morphology (high-turgor appearance, boutons with connectors), to the A-cells of the medium-resolution stack (Figure 3). Within these cells, an average of 8.1 synapses per cell is found in the reconstructed area; these are found primarily in the boutons. A-cells are predominantly presynaptic to R-cells and sometimes to D-cells. Furthermore, A-cells are sometimes postsynaptic to R-cells (Table 1).

## Discussion

The term ‘connectome’ refers to the mapping of all neural connections within an organism's nervous system or a confined part of it. These ‘wiring diagrams’ can be defined at different levels of scale, corresponding to levels of interest or the spatial resolution of imaging, for example, the microscale, mesoscale and macroscale [53]. A connectome at the macroscale (light microscope level) attempts to resolve different brain regions or neuropils and the pathways in between; these brain maps were established over the last hundred years for various species. These days with the help of various new techniques and increased computing power, the meso- and microscale (electron microscope) levels come into focus. At the mesoscale level, the morphology of distinct populations of neurons within a processing unit (for example, a column or a neuropil) is mapped. This level of analysis can be complemented by the microscale level, which involves mapping single neurons and their connectivity patterns (synapses), which according to Sporns *et al*. [53] will remain infeasible for an entire brain, at least for the near future. Recently, two ambitious scientific research projects, the Human Brain Project (by the European Union) [54,55] and the BRAIN Initiative (by the United States) [56,57], were launched to map these connection patterns in the human brain.

At the meso- and microscale levels, the basic architecture of sensory neuropils in both vertebrates (for example, the visual cortex in the human brain [58]) and invertebrates (for example, the optic lobes of the compound eyes in insects and crustaceans [43,59]) is characterized by columns and layers. The vertical columns, for example, in the insect lamina and medulla [11,12] are composed of repetitive subsets of afferent fibers (for example, those of the retinula cells) and characteristic postsynaptic neurons (for example, monopolar cells) that form the basic functional unit of a system (for example, visual system). Often, these columns are horizontally layered (for example, strata M1 to M6 in the medulla).

In the present study, we analyzed the pycnogonid visual neuropil at macro-, meso- and microscale levels to examine the principles that underlie this (simple) visual system and whether they compare to more complicated ones.

In the low-resolution stack, the macroscale observations of Lehmann *et al*. [49] can be confirmed. After entering the brain, the fiber bundle with the R-cell axons is split; one part of the axons ends in the first visual neuropil, and the other part passes the first visual neuropil and terminates in the second.

At the mesoscale level, aside from the R-cells, six different cell types can be distinguished in the first visual neuropil: five descending and one ascending cell type. The neuron gestalten are identified with two different approaches, providing support that both our three-dimensional-reconstruction and the Golgi-profiles give correct pictures of the neurons.

Three types of descending cells (D1, D2 and D3) are responsible for the subdivision of the first visual neuropil into two hemineuropils; these cells do not cross the border in between. In contrast, D4 neurons occur in both hemineuropils at once and provide lateral interactions between the two hemineuropils. The interpretation of the D5 cells is difficult. Here, these cells are found only in the right hemineuropil, which is most likely a sampling artifact, and the D5-cell bodies of the left hemineuropil are beyond the examined volume and, hence, are not reconstructed. At the microscale level, the D-cells are frequently postsynaptic to the R-cell axons and hence are second-order neurons. One individual R-cell is presynaptic to several D-cells and one individual D-cell is postsynaptic to several R-cells, indicating divergence and convergence. Concerning the synaptic pattern, no reliable separation between the five different D-cells could be made in the high-resolution stack. However, the reconstructed cells vary in the tangential size of the field they cover in a way that is analogous to their appearance in the medium-resolution stack, indicating that the synaptic pattern is similar in all descending cells.

The ascending neurons are higher-order neurons of a wider field throughout both hemineuropils. These cells are commonly presynaptic and sometimes postsynaptic to R-cells and, hence, play a feedback role in the system.

Furthermore, at the mesoscale level, it is observed that the first visual neuropil is split into two hemineuropils or columns. This is visible in both the SEM images and the three-dimensional reconstructions. The most plausible explanation of this subdivision is that one hemineuropil is linked to the anterior and the other to the posterior eye of the ocular tubercle. Additionally, in the two hemineuropils, at least three different layers of similar thicknesses are observable. In the upper third of the neuropil, the neurites of the unipolar cells enter the neuropil. Here, just a few collaterals were found. In the medium range of the neuropil, a number of things happen: most of the collaterals of the unipolar cells are found here, the branching and bifurcation of the D2 and D3 neurons occurs in this region, and finally the D4 neurons build here their tangential branches that reach into the two hemineuropils. Furthermore, in the medium range of the neuropil, which is analyzed at the microscale level in the high-resolution stack, additionally various synapses occur (whether and where synapses occur in the upper and lower ranges of the neuropil remains unclear at present because these regions were not studied at higher resolution). In the lower third of the neuropil, no more branching or bifurcation occurs, but numerous collaterals are found.

**Figure 8:**
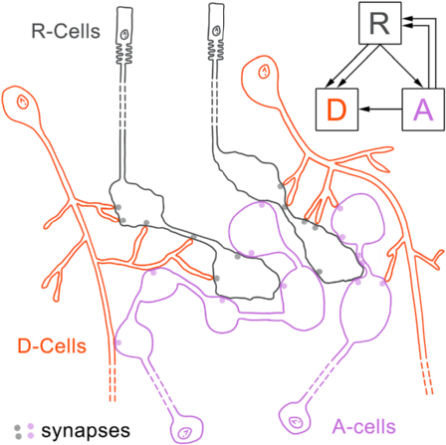
**Wiring diagrams of the major pathways in the first visual neuropil of the pycnogonid** ***Achelia langi*** **.** Pathways represented by >1 synaptic sites are shown. R-cells provide input into the system and are presynaptic to D- and A-cells. D-cells are predominantly postsynaptic to R-cells and, hence, are second-order neurons. A-cells are pre- and postsynaptic to both R- and D-cells and may play a feedback role.

This analysis reveals that the R-cells provide the input into the system, primarily on the D-cells. Because the D-cells rarely appear to be presynaptic in the first visual neuropil, these cells most likely synapse and, hence, integrate information to higher visual centers that were not identified in this study. These centers could be the second visual neuropil or the arcuate body, which in chelicerates is closely associated with the visual system [60]. The A-cells play a special role in this system, being pre- and postsynaptic to both R- and D-cells. Hence, these cells collect information from the input (R-cells) and the second-order cells (D-cells) but also circulate information back to these cells. Mechanisms such as lateral inhibition, contrast enhancement and other filter functions could be behind this feedback loop. Furthermore, principles of divergence in the R-cells and convergence in the D-cells are found. A summary of the visual pathways are given in the wiring diagrams in Figure 8.

A comparison of our findings with those in other arthropods proves to be difficult, as representatives of only a few taxa have been studied in sufficient detail to allow comparison of neuron morphology. Especially for median eye visual systems, just a few Golgi studies are available.

Hanström [61] reported for *Limulus* that neurites with cell bodies around the neuropil enter the median eye neuropil. Some of these neurites end in the arcuate body and some below the arcuate body. Clear statements on the morphology of these cells are lacking, but their position is the same as the descending unipolar cells found here. Strausfeld *et al*. [62] reported ascending broad field L-cells in the first median eye neuropil of *Cupiennius salei* (Araneae) that spread through a roughly circular area equivalent to several R-cells. By comparison, the ascending cells of *A. langi* also spread through wide reaches of both hemineuropils. The three-dimensional-EM study by Lacalli [63] of the larval nauplius eye center of the copepod *Dactylopusia sp.* is quite revealing. Here, the three eyecups of the nauplius eye are connected to the naupliar eye center. This neuropil is subdivided into three cartridges, each receiving R-cell axons from one of the three eyecups. Several second-order unipolar neurons (LR-cells) with cell bodies above the neuropil postsynaptic to the R-cell axons are found. Additionally, higher-order neurons (M- and E-cells) occur in the neuropil. A similar subdivision (two eyes, two hemineuropils) is found here in the first visual neuropil of *A. langi*. The morphology and synaptic pattern of copepod LR-cells is similar to that of the pycnogonid D-cells, but cells presynaptic to the R-cells, similar to the A-cells in pycnogonids, have not been identified.

The only arthropod visual system studied in great detail so far is that of the lateral compound eyes in some insect and crustacean species, namely three-dimensional-TEM of *Drosophila* [11–13,64], Golgi- and Golgi-EM-studies of insects [43,65–68], and Golgi- and Golgi-EM-studies of crustaceans [69–73]. The lamina's (that is, first visual neuropil’s) cell types are best characterized in the fruit fly *Drosophila melanogaster*, but the principles are similar in other insect species. The R-cells 1 to 6 provide input from each ommatidium and synapse to the lamina cartridges, the functional units of the lamina, which are composed of approximately 13 cells: the processes of five monopolar cells (L1 to L5), one or two amacrine cells, as well as three medulla neurons (C2, C3 and T1) and three glial cells. Additionally, two types of long visual fibers from the ommatidium, R7 and R8, pass the lamina and project to the medulla (second visual neuropil) [7]. In contrast, in crustaceans, R-cells 1 to 7 end in the lamina and R8 in the medulla. Here also, monopolar cells are found with similar characteristics as in insects. However, there is some disagreement about their number and nomenclature [69,74,75].

The synaptic organization in the lamina of *Drosophila* is studied and reviewed in detail by Meinertzhagen and O'Neil [7] and by Meinertzhagen and Sorra [11]. In the lamina, the R-cells are predominantly presynaptic to the monopolar cells L1 to L3 and to the amacrine cells. The L-cells in turn have only a few presynaptic sites (to R- and other L-cells) in the lamina. The amacrine cells are frequently presynaptic to R- and L-cells and often to T-cells. Finally, of the medulla neurons, only in C-cells do a few synapses occur, being presynaptic to L-, T-, and amacrine cells; T-cells are free of synapses in the lamina. All of these synapses are often multiple-contact synapses (dyads, triads, and tetrads). In the lamina of the crayfish *Pacifastacus leniusculus* the R-cells are also presynaptic to the monopolar cells [71].

## Conclusions

When comparing our results with the characteristics described in the compound eyes in *Drosophila,* we found striking similarities in the morphology and synaptic pattern of the visual neurons. The situation of the descending unipolar neurons in *Achelia* is similar to the monopolar cells in the compound eyes. Both have their cell bodies above the neuropil, each providing a single neurite that extends through the neuropil. In both, one can distinguish between cells that have collaterals in just one functional unit (that is, column in *Drosophila* or hemineuropil in *Achelia*; D1, D2 and D3 in *Achelia* and L1, L2 and L3 in *Drosophila*) and cells that provide lateral interaction between neighboring columns/hemineuropils (D4 in *Achelia* and L4 in *Drosophila*) and cells without or with very few collaterals in the first visual neuropil that contribute little to the neuropil organization (D5 in *Achelia* and L5 in *Drosophila*). Additionally, the synaptic pattern is similar. The D- and L-cells, respectively, are predominantly postsynaptic to the R-cells, and, hence, these cells are second-order neurons. Moreover, in both, these cells are rarely presynaptic to other cells in the particular neuropil. Contrary to these similarities, the morphology of the bifurcated D3 cells in pycnogonids has no counterpart in the compound eye lamina (Table 2).

**Table 2:** **Comparison of the synaptic patterns of the different cell types in** ***Achelia langi*** **and** ***Drosophila melanogaster*** **(** ***Drosophila*** **simplified after Meinertzhagen and Sorra** [11]**)**

**Studied species**	**Presynaptic cells**			**→ presynaptic to ↓**
	**R-cells**	**D-Cells**	**A-cells**	
*Achelia langi*	-	+	+++	R-cells
	+++	+	++	D-cells
	++	+	-	A-cells
	**R-cells**	**L-Cells**	**C-, T- and amacrine cells**	**→ presynaptic to ↓**
*Drosophila melanogaster*	-	+	++	R-cells
	+++	+	++	L-cells
	++	-	-	Amacrine cells

‘-‘ never; ‘+’ sometimes; ‘++’ often; ‘+++’ very often.

Furthermore, the ascending cells that integrate a wider field of the neuropil are found in both systems as well. In *Drosophila* there are three types of ascending cells (amacrine cells and the medulla neurons C and T). In *Achelia,* we found only one not specifically shaped type, but the synaptic pattern of these A-cells resembles the amacrine cells in *Drosophila*. In both species, these cells are frequently presynaptic to R-cells. However, the amacrine cells in *Drosophila* are often also presynaptic to T-cells from the medulla (Table 2). The medulla has no counterpart in the pycnogonid brain, and hence this cell type and such connections of the ascending neurons are not observed in *Achelia*.

Moreover, the synaptic pattern of the R-cells is the same. In both systems, these cells are predominantly presynaptic to the D- and L-cells, respectively, and frequently to the A- and amacrine cells, respectively, and are postsynaptic to the A- and amacrine cells, again, respectively (Table 2).

Finally, in both, the synapses between the different cell types are often multiple-contact synapses (dyads, triads, tetrads, or in pycnogonids even more).

Despite this high degree of correspondence, we think it would be premature to use the term homology for the correspondent cell types (D-/L-cells or A-/amacrine-cells) because only a few species have been analyzed at this level.

Although the pycnogonid visual system is morphologically and physiologically simple, and is found in a group that is positioned far away from insects and crustaceans in the arthropod tree, we found at least a foreshadowing of the principles described for the highly evolved visual systems of these groups. Already, rather than diffusely shaped neurons, distinct neuron types are found that can be characterized by their branching mode, dendrite length, width of the innervated field and their synaptic pattern. The second-order neurons have a distal cell body and descending neurites that are postsynaptic to terminals of the R-cells. These neurites form functional units (two hemineuropils comparable to the columns in insects and crustaceans), and their branches and collaterals at distinct levels make layers. Additionally, second-order neurons of a wider field are found that connect the hemineuropils, or rather, neighboring columns. And finally, higher-order feedback neurons with ascending neurites and branches that diverge to the wider field of the neuropil, being presynaptic to the R-cells, are found. Additional similarities with other arthropod median eye neuropils are found in pycnogonids. These are the position of the neuropil and the innervation pattern by the R-cells [49,76], as well as the subdivision of the neuropil, with each division responsible for one single eye and the presence of unipolar ascending and descending cells.

To put it in a nutshell, the connectome of the first visual neuropil of the pycnogonid *A. langi* has a well-organized architecture. It is composed of distinct cell types with characteristic synaptic patterns and already shows principles of the columns and layers design. Additionally, features of both chelicerate median and mandibulate lateral eyes are found in the pycnogonid visual system, indicating that these characters might be plesiomorphies and part of the ground pattern of the Euarthropoda.

## Methods

### Specimen collection

Specimens of *A. langi* (Dohrn, 1881) (Ammotheidae) were collected for FIB-SEM during field trips in May 2011 to Rovinj (Croatia). Specimens of *A. vulgaris* (Costa, 1861) were collected for the Golgi technique during field trips in 2009 and 2010 to Rovinj. Species were determined following Dohrn [77] and Bamber [78].

### Focused ion beam/scanning electron microscopy

After dissection of the abdomen, legs, and proboscis in 4% glutardialdehyde in 0.1 M cacodylate buffer at 4°C, the animals were fixed in 4% glutardialdehyde and 1% tannin in 0.1 M cacodylate buffer at 4°C and stored under refrigeration at 4°C. After transportation to the laboratory in Munich, the specimens were osmicated in 1% OsO_4_ in 0.1 M cacodylate buffer for two hours at 4°C. To enhance contrast, specimens were *en bloc* stained with 4% uranyl acetate for one hour at room temperature. After dehydration in a graded acetone series, the specimens were embedded in epoxy resin (Glycidether 100; two days at 60°C and one day at 90°C).

#### Low-resolution stack (transversal view)

To approach the visual neuropils, the specimen was trimmed transversally with a diamond knife on an RMC-MTXL ultramicrotome until just before the visual neuropils appeared. After trimming of a cuboid-shaped ‘mesa’ containing the pycnogonid brain with a glass knife [79], this mesa was removed from the epoxy block and mounted on an aluminum stub covered with a thin layer of unpolymerized epoxy resin as glue. The transversal block face was now oriented vertically on the stub, allowing transversal milling of the left neuropils by the FIB. After polymerizing the epoxy resin (one day at 60°C), the stub was coated with carbon with a Balzers High Vacuum Evaporator BAE 121 to make it conductive.

The sample was milled and imaged with a Zeiss Auriga CrossBeam Workstation (Carl Zeiss Microscopy, Oberkochen, Germany). For slicing, the conditions were as follows: 500 pA milling current of the Ga-emitter; with each step, 10 nm of the epoxy resin was removed with the focused ion beam. SEM images (2,048 × 1,536 pixels) were recorded from every third slice at 1.5 kV, resulting in a stack of 682 grayscale images (voxel size 32 × 32 × 30 nm; total volume: 65.5 × 49.2 × 20.5 μm).

#### Medium-resolution stack (frontal view) 

The specimen was prepared and imaged as for the low-resolution stack, with the only difference being that the specimen was trimmed frontally to allow frontal milling of the left first visual neuropil by the FIB. With a milling rate of 5 nm (every third slice recorded), an image stack with 1,031 planes was acquired (voxel size 12 × 12 × 15 nm; total volume: 24.6 × 18.4 × 15.5 μm).

#### High-resolution stack (frontal view)

The same specimen was used as for the medium-resolution stack. The medium range of the contralateral right first visual neuropil was imaged with FIB-SEM with a milling rate of 5 nm (every third slice recorded, 212 images; voxel size 6 × 6 × 15 nm; total volume: 12.3 × 9.2 × 3.2 μm).

### Image editing and three-dimensional reconstruction

The images were contrast-enhanced and sharpened using unsharp masking in Adobe Photoshop® CS5 (Adobe Systems), then aligned, manually segmented and surface rendered in Amira® 5.2.0 (Visualization Sciences Group).

In the medium-resolution stack, the profiles of a representative ensemble of 34 cells were reconstructed. In the high-resolution stack, the profiles of a representative ensemble of 33 cells were reconstructed and presynaptic sites of 95 chemical synapses are localized on the basis of synaptic vesicles. Care was taken that cells postsynaptic to the reconstructed cells were selectively reconstructed as well.

The interactive supplement figure was created following Ruthensteiner and Heß [80] with updated software.

### Golgi technique

The abdomen, legs, and proboscis were dissected and the cuticle regions surrounding the central nervous system were perforated to increase the probability of staining the desired areas. The preparations were submitted to two cycles of the Golgi-Colonnier method [81], embedded in epoxy resin and sectioned (10 to 20 μm).

### Data access

The profiles of all reconstructed cells are uploaded to Morph D Base [82]. We will also provide original data upon request. The requesting party will need to supply a hard drive.

## Additional file

Additional file 1:
**Interactive supplement Figure: Lateral view of three-dimensional reconstruction of visual neuropil 1. A**, all reconstructed cells of all six neuron types shown. **B**, A1 neurons omitted thus subdivision of the neuropil becomes visible; note D5 neurons mainly in right hemineuropil. **C**, A1 and D5 neurons omitted; note D4 neurons occur in both hemineuropils at once. **D**, A1, D4, and D5 neurons omitted; note D1, D2 and D3 neurons build two hemineuropils. **E–J**, distribution of different cell types (separately) within neuropil. The interactive three-dimensional-model can be accessed by clicking into the figure (Adobe Reader Version 7 or higher required). Rotate model by dragging with left mouse button pressed, shift model: same action + ctrl, zoom: use mouse wheel (or change default action for left mouse button). Select or deselect (or change transparency of) components in the model tree, switch between prefab views or change surface visualization (for example, lightning, render mode, crop and so on.).
