# Supplementary material for: Dissecting a neuron network: FIB-SEM-based 3D-reconstruction of the visual neuropils in the sea spider Achelia langi (Dohrn, 1881) (Pycnogonida)
Source: BMC Biol. 2014 Aug 13;12:59. doi: 10.1186/s12915-014-0059-3 (PMC4159573; doi:10.1186/s12915-014-0059-3)
Supplement: Additional file 1: — Interactive supplement Figure: Lateral view of three-dimensional reconstruction of visual neuropil 1. A, all reconstructed cells of all six neuron types shown. B, A1 neurons omitted thus subdivision of the neuropil becomes visible; note D5 neurons mainly in right hemineuropil. C, A1 and D5 neurons omitted; note D4 neurons occur in both hemineuropils at once. D, A1, D4, and D5 neurons omitted; note D1, D2 and D3 neurons build two hemineuropils. E–J, distribution of different cell types (separately) within neuropil. The interactive three-dimensional-model can be accessed by clicking into the figure (Adobe Reader Version 7 or higher required). Rotate model by dragging with left mouse button pressed, shift model: same action + ctrl, zoom: use mouse wheel (or change default action for left mouse button). Select or deselect (or change transparency of) components in the model tree, switch between prefab views or change surface visualization (for example, lightning, render mode, crop and so on.). [file 12915_2014_59_MOESM1_ESM.pdf]

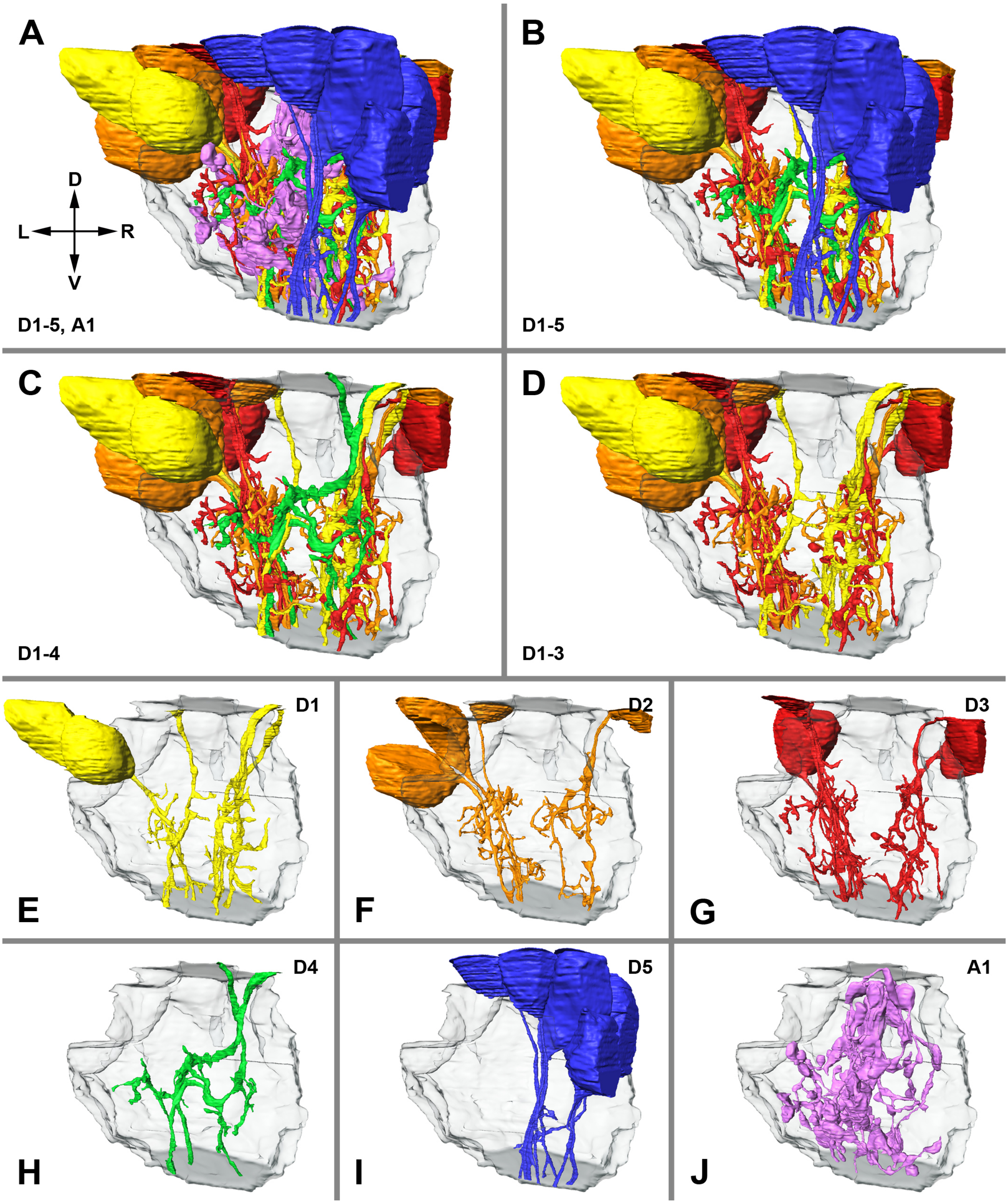

Interactive supplement Figure: Lateral view of 3D reconstruction of visual neuropil 1.
A, all reconstructed cells of all six neuron types shown. **B**, A1 neurons omitted thus subdivision of the neuropil gets visible; note D5 neurons mainly in right hemineuropil. **C**, A1 and D5 neurons omitted; note D4 neurons occur in both hemineuropils at once. **D**, A1, D4, and D5 neurons omitted; note D1–3 neurons build two hemineuropils. **E–J**, distribution of different cell types (separately) within neuropil. **The interactive 3D-model can** be accessed by clicking into the figure (Adobe Reader Version 7 or higher required). Rotate model by dragging with left mouse button pressed, shift model: same action + ctrl, zoom: use mouse wheel (or change default action for left mouse button). Select or deselect (or change transparency of) components in the model tree, switch between prefab views or change surface visualization (e.g. lightning, render mode, crop etc.).
